# Access to Interaction and Context Through Situated Descriptions: A Study of Interpreting for Deafblind Persons

**DOI:** 10.3389/fpsyg.2020.573154

**Published:** 2020-12-14

**Authors:** Eli Raanes

**Affiliations:** Department of Teacher Education, Faculty of Social and Educational Sciences, Norwegian University of Science and Technology, Trondheim, Norway

**Keywords:** participation, environmental description, deafblind, interpreting, tactile sign language, haptic signals, multimodality, interaction

## Abstract

This article focuses on how to provide environmental descriptions of the context with the intent of creating access to information and dialogical participation for deafblind persons. Multimodal interaction is needed to communicate with deafblind persons whose combined sensory loss impedes their access to the environment and ongoing interaction. Empirical data of interpreting for deafblind persons are analyzed to give insight into how this task may be performed. All communicative activities vary due to their context, participants, and aim. In this study, our data are part of a cross-linguistic study of tactile sign language and were gathered during a guided tour for a deafblind group. The guided tour was tailored to a specific group (adult deafblind tactile signers and their interpreters) visiting one of the oldest cathedrals and pilgrim sites in Scandinavia, with interpreters following up the guide’s presentation and providing descriptions based on the given situation. The tour and the interpreters’ work were videotaped, and the ongoing interaction and communication have been studied through video-ethnographic methods and conversational analysis. The data have been investigated for the research question: What elements are involved in descriptions to provide deafblind individuals access to their environments? Theories from multimodality communicative studies are relevant for the ways tactile descriptions are presented and analyzed. Some of this is an investigation at a microlevel of interaction. An overall inspiration for this study is interaction studies with data from authentic formal and informal conversations and ways of analyzing embodied action and situated gestures in studies of human interaction. Also, concepts of “frontstage,” “backstage,” and “main conversation” are brought into our interpreter-mediated data to follow the role of building meaning in complex conversations. Theories on interaction are used in the analyses to illustrate the participating framework between the guide, the interpreter, the deafblind person, and the situated frame of their interaction. The study opens for a broader understanding of the repertoire of multimodal interaction and how such interaction may be handled as inputs in communication processes. This is of relevance for communication with deafblind persons, for professionals meeting blind and deafblind clients, and for knowledge of multimodal interaction in general.

## Introduction

### Description as Part of Interpreters Work

Deafblind persons’ sensory loss varies in degrees from having some or no residual sight or hearing (Petren, 1980; Möller, 2008; Creutz, 2019). The way descriptions are performed depends on the deafblind person’s needs and mode of communication. Educational programs for interpreters for the deafblind have environmental descriptions as one of their subjects. This study’s data are interpreter-mediated action where the certified interpreters understand that access to interaction depends on more than access to the spoken words. Interpreter-mediated interaction for deafblind people consists of these three main tasks: (1) translate spoken and signed messages in an interpretation process involving different languages and language modalities, (2) describe the environment and the context of the communication settings, and (3) guide the deafblind person finding their way during the interpreted event based on personal communication and guiding needs. From 2013, this has been an understanding of the interpreter role described in the *Deafblind Guidelines for Interpreter Education*, addressed by the World Association of Sign Language Interpreters (The World Association of Sign Language Interpreters [WASLI], 2013). These job guidelines follow the definitions and curricula for trained interpreters’ duties by Scandinavian education programs, including training and qualifying interpreters to be both sign language interpreters and interpreters for deafblind clients.

This qualitative study aims to reveal by video analysis structures used in communication with deafblind people where environmental description is needed. A small set of selected data is analyzed and described in detail, with the aim to contribute to more insight and discussions about some specific communicative practices and interaction settings.

The public welfare systems in the Scandinavian countries provide interpreter services free of charge, and interpreters may be used in both public and private settings when needed (Erlenkamp et al., 2011; Berge and Raanes, 2013), making description a relevant task in many different interpersonal situations. During the last decades, the deafblind community has increasingly used interpreter services in their daily activities, cultural events, and work-related assignments (Hjort, 2008; Agenda Kaupang, 2016). Interpreting spoken/signed messages and providing descriptions are both important parts of enabling the deafblind person to have access to and participate in the given interaction and context. When the amount of information is large, a selection of information and ways of providing the information must be taken into account according to what is relevant for the uniqueness in the communicative setting. There are a wide range of settings where deafblind individuals order an interpreter service and a wide range of situations where such ethical decisions are discussed (Ministry of Labor and Social Affairs/Agenda Kaupang 2016). The tasks related to the description for deafblind people entail responsibilities for those working professionally as interpreters, related to the client’s self-determination and power. These aspects are part of an ongoing debate within the services working with deafblind people (Raanes, 2018), as well as within the deafblind communities working on developing new conventions to convey environmental information (Nielsen, 2012; Palmer and Lahtinen, 2015; Edwards, 2017).

### Situated Actions

All interaction and communication are situated in a context influenced by the participants’ relation and understanding of the ongoing situation. This makes communication situations complex and grounded in an interactive and mutual process among those taking part. In situations where there is a feeling of communicative flow, we can adjust our communication to those we interact with. We establish ways of clarifying and handling processes as building on others’ inputs, regulating how we take our turns in the conversation, how and whether we give feedback to what is said, and how to introduce or follow up communicative actions (Sacks et al., 1992; Linell, 1998). These are important skills and inputs to build up various communicative events. Previous experience, general knowledge, and competence in language and genre help us take part and adjust our communication in dialogical actions. In Goffman’s theories, human interaction is seen in a dramaturgic perspective where the interaction is dependent upon time, place, and audience. In other words, the immediate scene leads to how we present our self and interact with others in the shifting scenes of everyday life (Goffman, 1959). In a conversation among participants, the interaction may appear in simultaneously differentiated layers involving the main conversation and partly also in various ways of acting related to this main conversation. In Goffman’s terms, we perform “frontstage” or take part in parallel conversations involving clarification and sequences of inputs “backstage” at the scene of the interaction. The signals used to maintain the conversations are both manual and non-manual inputs to the dialog and are part of establishing and constructing the communicative process as situated, sequential activities (Goodwin, 2013). When and how to interact, and when to listen and observe, are part of our understanding of context and our communication skills (Linell, 2009). Multimodal perspectives on conversations have changed the understanding and the way of analyzing naturally occurring conversations, where artifacts and bodily orientation are inputs to the interaction during conversations and affect the meaning-making processes (Goodwin, 2000; Mondada, 2009, 2014, 2016). Studies in this CA tradition (conversation analysis of video-recorded multimodal discourse) are approaches that are relevant to this study, studying communicative events where tactile sign language is in use.

### Situated Descriptions

Descriptions must be situated to make meaning. A deafblind person taking part in a meeting or a discussion must know about the context and the purpose of what is happening, since much of environmental information is provided with auditive and visual clues not accessible to persons with a dual sensory loss. When taking part in a discussion, it is important to know who is talking, to whom, with what intention, and with what response. Interpreters need to have an awareness of how context and actions are part of the communication situation (Berge and Raanes, 2017; Raanes, 2018). Even if all that is said is interpreted to a deafblind person, if there is no description of the context or the participants’ actions, the meaning of the interpreted word may not be understood. Two main concepts contribute to the choices interpreters make while building up descriptions – the description must be connected to *a situated understanding* of what description is needed, and the selection of information has to provide *critical information* in the situation (Raanes, 2018). When time is limited or several things happen at the same time, choices must be made on how to provide the critically needed description. The amount of selection and the organization of what is described are crucially important. Information that is not framed in an understanding of the context may be difficult to follow. The situated environmental description requires a focus on preparing the deafblind person to make his or her own choices. To do so, you need access to what is the critical information required to participate in the given situation (Berge and Raanes, 2013). Descriptions must be provided in the knowledge that there is a risk in giving incoherent or excessive information that may obstruct participation and prevent the deafblind person from reading the situation (Willoughby et al., 2014; Raanes, 2018; Creutz, 2019). Goffman; Goffman’s (1959; 1974) theories on how we in interaction play out roles in various scenes of our ordinary life, and on the complexity of how we may change between perspectives from what to focus on, have enhanced our understanding of interaction. Activities following the ideas of his theories may also be seen in interpreter-mediated interactions and in the ways interpreters work to build up knowledge about the scene, the action, and the participants. This can be observed when interpreters change position and/or their movements to indicate the direction of those who have the floor in a conversation, as described by a variety of embodied actions, to address the target of meaning construction (Raanes, 2018).

The ways guides communicate to situate their stories in areas as museums and historical sites are discussed in Mellemsether and Müller (2016). They explore the professional adjustments done in such settings, and how access to the sites is supported by guides having skills to adjust to different groups, e.g., deaf or blind guests or children, etc. Interactions in exhibitions and museums are discussed in terms of being multimodal expressions by Meisner et al. (2007).

### Making Something Common

The word “communication” stems from the Latin verb *communicare*, which means “to make something common.” Taking part in communication includes a relationship with someone to interact with and a joint focus on something. This focus may be on something in the present situation or on something abstract that is not part of this situation. Mastering the shifting focus during a conversation, and getting access to those shifts, is an essential part of communication skills, and the same goes when a person takes part in mediated conversations (Rommetveit, 1974; Trevarthen, 1998). A dual sensory loss challenges the deafblind person’s access to having situated information from the common situation being shared, so that description of the ongoing situation is needed to provide information and access. It is important to provide descriptions based on an ethical reflection of each person’s individual opinions and choices, and interpreters do not aim to “explain” what is happening but to stimulate interaction in the communicative setting based on the deafblind’s preferences and own understanding in the situation (Raanes, 2018).

### Earlier Research

When interpreting for the deafblind became a professionalized skill, *description* became a subject in the curricula. In Swedish and Norwegian programs, the subjects were from the early 1990s based on experience-based compendiums building on experiences by interpreters describing their approach to this topic. Research-based knowledge of environmental description has for many years been rare. International studies on this skill remain limited.

Fieldwork in interpreting has painted a complex picture of what it means to perform interpreting and communicate *via* an interpreter in public service institutions, where healthcare, social services, legal issues, and other matters are dealt with. Models of dialog interpreting that regard turn taking as independent textual units have failed to account for the contextualized dynamics of interpreted talk. Research on interpreting “as interaction” has highlighted that interpreted conversations are tightly linked to activities in which the participants’ contributions are attributed to meaning and purpose. An increased need for quality interpreting in the public sector has triggered research in the area of training, teaching, and learning, revealing both a need and a renewed possibility of better professionalization (Wadensjö, 1998; Llewellyn-Jones and Lee, 2014; Napier et al., 2018).

In this study, dialogism provides the theoretical approach for understanding description rooted in the interaction in a situation. This approached is widespread in studies of communication and interpreter-mediated communication (Linell, 1997; Wadensjö, 1998). Conversations are understood as a human interaction that is performed in a situated and sequential manner, and where meaning-making processes are built on negotiation during the talking process. The sum of visual and auditive information surrounding deafblind persons is potentially huge, and an environmental description must be focused on a reflection of what is critical information in the given situation (Raanes, 2018, p. 205).

Communitive interaction is a collaborative project that requires teamwork and cooperation. Because of the time needed to process the interpretation, interpreter-mediated communication makes collative aspects even more important by the participants taking part in the situation. All situations differ, depending on the uniqueness of the expectation, the purpose of the event, the relation, and the time available.

Berge and Raanes (2013) have analyzed a collaborative understanding in the interaction process in naturally occurring interpreter-mediated group discussions between deafblind participants. The analyzed material shows how formal group discussions in interpreter-mediated meetings depend on precise and simultaneous environmental descriptions. The interpreter’s action plays along with the understanding of the scene and role of the participants and the situation, following Goffman; Goffman; Goffman’s (1971; 1974; 1981) concepts of framing talk and interaction.

The translator principle by Hieronymus (lived 347–420, and known from his translation of the Bible to Latin) is *non-verbum e verbo sed sensum de sensu*, “do not translate word by word, but meaning by meaning” (Robinson, 2002, p. 25). This is demonstrated in research in general, also in interpreting for deafblind persons. Frankel (2002) has studied how negotiation was translated when interpreters were working from visual ASL (American Sign Language) into tactile ASL, and found that the interpreters not only focused on the format of translation of the words/signs of negotiation. The interpretation process was about how to make the text available for the deafblind person, and by doing so, the interpreters chose to change some of the utterances’ structure to make the meaning accessible (Frankel 202, p. 169). In a study by Metzger et al. (2004), data from interpreters working into tactile ASL showed how the interpreters chose to add self-generated utterances if needed, to make the meaning in the message come through. Metzger et al.’s results were based on examples from interpreting in different communication situations (classroom situation, medical interview, and panel discussion). Raanes (2018) has studied video recordings of interpreter-mediated conversations in naturally occurring events of daily activities, analyzed according to the content and timing of environmental description. Interviews by the participants (interpreters and the deafblind clients) were also recorded and analyzed as part of the study. This study analyzed not only texts spoken/signed in the situations but also how access to environmental descriptions was handled in the interaction. The findings introduce three principles of organizing descriptions for deafblind people, namely that interpreters should provide an overview, offer a critical selection of details, and be aware of and adapt to a dialogical frame (Raanes, 2018, p. 209).

Haptic signals (informative touches on the deafblind person’s arm, shoulder, and back) and more awareness of tactile assignments are important tools that provide a new understanding of how to gain access to and participate in interpreting situations for deafblind people (Lahtinen, 2003; Skåren, 2011; Bjørge and Rehder, 2015). The deafblind communities themselves emphasized the importance of description, and in the Scandinavian countries, research has been carried out on how haptic signals and bodily signals would benefit in the process of getting access to communicative settings. According to Lahtinen, Palmer, and Lahtinen, “description supports actions and choices of a person with visual and dual sensory impairment and facilitates contacts with the environment” (2010, p. 3).

In the United States, deafblind societies have been involved in a pro-sign movement (Edwards, 2014). In the larger communities of deafblind people, this has established practices where awareness of involvement and direct access is emphasized.

Multimodal research and video-ethnographic studies motivate the investigation of real-life conversations in naturally occurring data (Knoblauch et al., 2006; Broth et al., 2014). Research on tactile sign language has provided new insight into the deafblind communities’ conventionalized language practices (Holmström and Mesch, 2018). Research on deafblind communication has shown how tactile modality leads to solutions that are unique to tactile sign languages, as when interlocutors use their own body as well as the interlocutor’s hand/body to express verb constructions through movements and positions (Raanes, 2006; Mesch et al., 2015). In the recent journal of *Frontiers in Education*, Gabarro-Lopez and Mesch (2020) analyze parts of the corpus data also in use for this article. Their findings demonstrate the variety of strategies in use to convey environmental information to deafblind persons in interpreter-mediated activities.

## Materials and Methods

The data for this study are part of a cross-linguistic corpus collection of tactile sign language from Norwegian and Swedish participants (Raanes and Mesch, 2019). The empirical data are language use collected in naturally occurring situations, in a research design following criteria and guidelines for qualitative research (Tong et al., 2007). The research team recruited informants who were fluent signers diagnosed with deafblindness and who use tactile sign language as their main method of communication. The informants were selected from active members in national organizations for the deafblind. A criterion for participation was a willingness to accept joining a research project which aimed to build more knowledge and to start a corpus base of tactile sign language use – this involved video recordings of conversations and interpreted activities. We were looking for four informants (two males, two females), and the project was presented to four prospective deafblind informants, *via* web-based reading programs accessible to deafblind persons. The data collection involved the participants traveling a long distance and taking part in a 3-day cultural event with varied activities and discussions designed and planned for deafblind participants. The first four informants we contacted were all willing to participate and to take time to join the scheduled days for the event. These informants themselves contacted their interpreter services and made arrangements with the interpreters they wanted to be involved with during travel and the event. This ensured involving interpreters preferred by the deafblind participants. The group of four deafblind informants (three women, one man) had the mean age of 59, ranging from 50 to 76. All had long experience in sign language usage and had, due to increasing sight problems, switched to using tactile sign language. Altogether, eight interpreters were part of the event, all women and experienced with interpreting for deafblind people. Due to the program’s length and intensity, there was a need for two interpreters for each deafblind person. All the interpreters were directly contacted by the researchers and were introduced to the research project and with the plan for recordings in advance. They agreed to participate in the study. When arriving at the site of the event, all participants were asked to give their informed approval before the program started. The relevant information was made available both in braille and in print and was presented directly to each participant following standards for research approval by the Norwegian Centre for Research Data (0000) (NSD project 192998).

Various activities were carried out during the 3 days of data collection. The total amount of data for the corpus collected during the event included close to 27 h of video recordings. For the present study, one delimited activity is being analyzed – a guided tour to a historical site. The chosen activity is from a specially tailored tour of the eleventh-century Nidaros Cathedral in Trondheim, Norway, a popular pilgrimage site in Northern Europe. The guided tour was planned by the church guide service and specifically prepared for deafblind participants. Several tactile historical attractions in the cathedral were included on the tour, which focused on the cathedral’s history, architecture, and pilgrimage tradition. The video material from the guided tour was selected as it should be from an event where description clearly was needed and had a distinct beginning (coming to the event) and ending (finishing the event and leaving the site). The guided tours were carried out twice, once for a Swedish group and once for a Norwegian group. For each deafblind person, two interpreters worked together in shifts during the guided tour. Besides the deafblind persons, the interpreters, and the guide, five researchers and research assistants were involved in the tour to make video recordings. The interpreter-mediated interactions were filmed with several cameras – following the activities from different camera angles – to produce video data of high enough quality for analytical purposes. The videotaped interaction in the cathedral analyzed for this study amounts to 274 min of recordings.

Entering such a site as a cathedral for a deafblind group may offer little information when you do not see or hear – even with a well-prepared guide. Two of the informants were able to use very restricted sight, like to see light coming in from windows and see shadows of persons moving in their field of vision. But the situation (taking part in a guided tour) is a typical environment where mediated information is needed – a site requested and called for description by the interpreters to make information accessible and interaction with the guide and the group possible.

These data are analyzed and will be presented in various formats, such as selections of annotations, photos, drawings, and summarized cases or narratives to illustrate interactive episodes. Those varied presentations are a way of illustrating environmental descriptions, where complex multimodal expressions of descriptive action are made accessible in text. For some of the examples, the transcripts are indeed detailed in order to describe the movements and responses. In some of the transcripts, the analytic focus is on a summary of the content of the descriptive performance, where the annotation texts in written form present a step-by-step description of the multiple interactions. As a qualitative study of description, turns involving interaction between the interpreters and the deafblind person were of specific interest. For some of the annotation forms, this represents an analytical process, where the video recordings are transcribed in great detail with the annotation tool ELAN. Here, the video pictures are linked together with transcriptions of precise annotations of what the interpreters, deafblind persons, and the guide did and what they communicated, with spoken words, signs, haptic signals, and movements all being studied – using one hand, both hands, bodily orientation, etc. For other parts of the process being analyzed, the findings are based on a conversation analysis that follows the meaning-making process in the interaction turn by turn. Analyzed extracts of actions are presented in the result and discussion part where the reports are made available through texts, transcripts, pictures, and drawings – all named and presented as *tables*.

The material will be analyzed qualitatively in order to answer our research question: What elements are involved in descriptions to provide deafblind individuals with access to their environments?

## Data Presentation and Analyses

Examples from the analyzed material from the interpreter-mediated guided tours of Nidaros Cathedral will be divided into four strategies for environmental description seen in the data:

1. The repertoire of multimodal communicative tools.
2. Topicalization.
3. Dialogical approach.
4. The impact of space and bodily orientation.

### The Repertoire of Multimodal Communicative Tools

The study of the videotaped data shows a continuous use of varied multimodal ways of communication through the guided tour. This variety of repertoire is shown in what the interpreters, the deafblind guests, and the guide do when describing and exploring the site. When providing descriptions, the interpreters use a mix of tactile signing, fingerspelling, writing in the person’s palm, haptic signal gestures, pointing, sensitive hand guiding, artifacts, and various bodily orientations and moves. The interpreters choose among these different multimodal communicative tools in their descriptions, depending on the context, what they want to address, and the response from the participants.

The transcript in Table 1 illustrates the variety of this repertoire in a sequence of the tour. The context of the transcribed sequence is that the guide has given some information about a silver cross halfway up the cathedral floor, and now she continues to walk further into the cathedral. The interpreters and the deafblind guests follow her, and they all stop in the middle of the cathedral. As the guide looks toward one of the deafblind guests (DB1), she lifts a small cross from her hood, representing the cathedral’s cruciform shape, and the interpreters immediately start their description. The transcript’s left colon details the communication and interaction between *the guide*, *the interpreter* (I), and *the deafblind guest* (DB1). The interaction is written in brackets, while signed utterances are translated into written, English sentences. The middle part of the transcript shows the tactile signs being used, written in capital letters according to the convention of sign language studies. The right part of the transcript marks the multimodal variations of communication.

**TABLE 1:** Transcript with variations of multimodal communication (D2-K3-067: 00:33–00:59).

	Communication and interaction	Signs/words	Multimodal variations
01: I	(The interpreter walks closely toward the guide, who stands in the center point of the cathedral holding a cross model and looking toward them)		Moving close to the guide; bodily orientation; eye contact with the guide

02: I	Here there is something (the sign SHOW is made toward the guide, and hands lead in the signing space toward her)	HERE NOW SHOW	Tactile signing; bodily orientation; variation in signing space

03: Guide	(Comes close to DB1 and lays the small cross in the person’s palm)		Awareness by proximity; presenting artifacts

04: DB1	(Holding and touching the shape of the cross in his hands)		The tactile orientation of artifact

05: I	(Points to the middle part of the cross model with her index finger, then takes DB1’s index finger and guides it to the middle part of the model)		Pointing; hand guiding

06: DB1	[Follows the interpreter’s hand-guided movement (explores the pearl in the center of the model)]		Interaction through movements; tactile exploration by touch

07: Guide	We are now where there is a pearl on the model.	(Talking)	Talking

08: I	([Tapping several times on DB1’s finger, now placed in the middle of the cross movements])		Haptic response signal

09: Guide	(The guide gets the model back from DB1)	(Talking)	Orientation; action

10: I	This area is here.	AREA HERE IN-THE-MIDDLE	Tactile signing

11: DB1	Yes, this spot here.	THIS SPOT YES	Signing; nodding; voice

12: I	Just in the middle.	M-I-D-D-L-E HERE	tactile fingerspelling; tactile signing

13: I	(Looking up to the ceiling)		Bodily movement and head movements tilting upward

14: I	Up here.	POINTING UP	Pointing (pointing with index finger high up in signing space)

15: I	The chandelier.	FORM-OF-OBJECT-HIGH-UP	Gesturing/signing the form with one hand

16: I	We see high up a huge chandelier.	LOOK CHANDELIER	Signing with both hands

17: I	c-h-a-n-[d] (fingerspelling)	C-H-A-N-[D]	Tactile fingerspelling; two-handed alphabet

18: DB1	(DB1 interrupts the fingerspelling) [How far] up is it, is it many meters?	[MUCH] HOW MANY METERS UP?	Signing; turn taking – letting go of physical contact with the interpreter’s hands

19: I	(Moving her body slightly toward the guide and translate DB1’s question) How many meters is it up to the roof?	(Talking)	Orientation; keeping hands still in upper signing space waiting for a response; upper body oriented toward the guide

The conversation sequence transcribed in Table 1 shows the many shifts and variations of communicative tools used to describe and give information about the part of the cathedral where they are located. Table 1’s transcription runs for 26 s of conversation and includes tactile signing, tactile fingerspelling, pointing, nodding, gesturing, hand guiding, variations in signing space, response signals by tapping the other’s hand, bodily orientation, and active use of artifacts to present the location through a cross that indicates the cruciform shape of the cathedral. All this varied use of communicative tools occurs naturally in the interaction and changes according to what seems efficient in the situation. Conversational analysis indicates that the participants understood what was being conveyed and that the communication flowed naturally. Their interaction and the turn shifts are followed up by relevant response building on to previous turns.

Through their description in the cathedral, we observe that all the interpreters use variations of a wide array of multimodal communication, as shown in Table 1. This finding points to the use of situated and embodied action used in addition to language (signs/words) to make access to the present environment. The *intentional ways the interpreters use their own body and movements* are through all the data’s examples seen when they are presenting mediating information about the environments. When leading and walking together with the deafblind person, the interpreter’s reflective bodily movements are done in various multimodal ways. Later in this study, we will return to examples of bodily movements as tools in descriptions, and we will refer to Table 1 when looking into further details in the interaction seen in this transcript.

### Topicalization: From an Overview Down to Details

**FIGURE 1:**
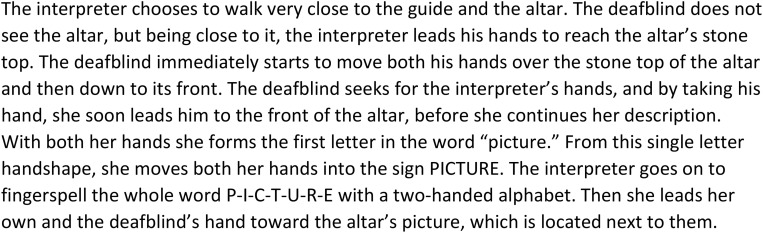
Transcript of the description presenting a new theme.

Knowing what is in focus is central to make meaning of mediated information. A precise description of details may have no value if mediated without a clear context or an awareness of what kind of interaction or situation it relates to Raanes (2018). In the data for this study, we see interpreters presenting an overview before describing details – when the topic changed during the guided tour, the interpreters followed this up by giving a brief presentation of the next topic to focus on. Introducing a topic may also be done by giving the deafblind access to a direct exploration of something representing the topic. In the transcript below (Figure 1), the guide leads the group to an altar, assumed to be the space where the reliquary of Saint Olav, in whose memory the cathedral was built, has rested for centuries. This represents a new post on the guided tour. The guide places herself close to the altar and calls for her visitors to come closer. What is most characteristic about this altar is the front, where a copy of an old painting formed as a cartoon of the saint’s life is seen (Figure 1).

By adding pictures of this performance into the transcript, we will investigate the details of the way the interpreter chooses to establish the topic, as based on the guide’s information:

The transcript in Table 2 shows in detail how the new post in the guided tour is introduced and made clear. The transcript begins when the deafblind explores the altar with his hands. The interpreter’s description starts when the deafblind’s hands touch what the guide has just started to talk about – a painting of the saint whose coffin once had been placed at this altar. The interpreter interacts and takes his hand to describe what he touches. As seen in the annotation, this sequence of descriptions also ends with the interpreter leading the deafblind down to touch the altar’s front, where the painting in question is. She thereby makes a connection and clarifies where the described painting is located.

**TABLE 2:** Transcript of the introduction to the decorated altar.

01	The interpreter takes the deafblind’s hand and lightly taps their hands on the front of the altar	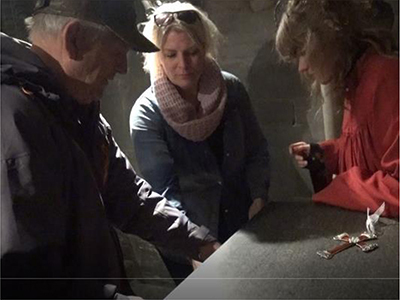	(The deafblind person on the left, interpreter in the middle, guide in red robe to the right)
02	With both hands, she forms the first letter of the word “picture” *	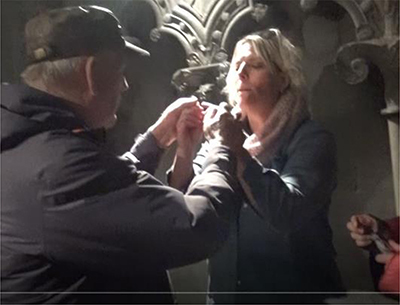	*(In TNTS, done with two-handed alphabet “B”)
03	From the handshape of forming the single letter – moving both hands into the sign PICTURE *	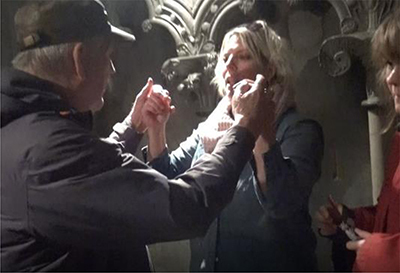	*The sign for PICTURE is done with both hands outlining a square – an iconic manual form that refers to several different objects and signs
04	Continues to fingerspell, letter by letter, the whole word P-I-C-T-U-R-E	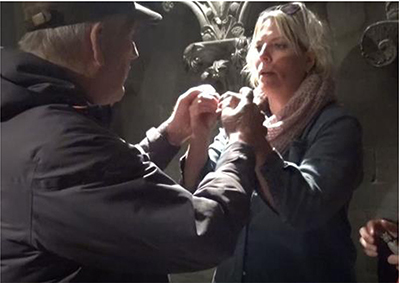	
05	The interpreter leads her own and the deafblind’s hand toward the altar’s picture, located next to them	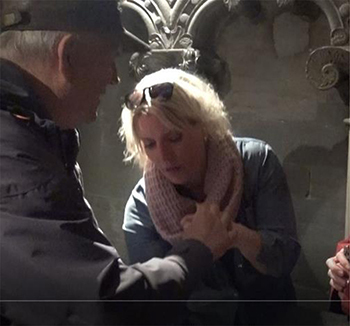	

During just 7 s, the annotation (in Table 2) follows in detail the interpreter’s coordination of various shifting communicative activities – hand guiding, use of hand alphabet, tactile signing, fingerspelling, and again hand guiding. During this time, the topic is made clear and the panting’s location is marked. Then (after the transcript above), the interpreters and the guide wait for the deafblind to move his hands to lightly touch the framed altar and the painting. With light hand movements, his exploration of the altar goes on for 14 s. They wait for him to do this and to raise up, searching for the interpreter’s hands. And then the guide starts presenting the tales about the saint’s life and the cathedral’s pilgrimage tradition. Being one of the main parts of the guided tour, this lasts about 9 min.

In this example, the interpreter chose to start her description by leading the deafblind’s hands so he could touch the altar before she named a keyword for the coming information – “picture” – referring to the copy of the old painting at the altar. With this deliberate choice of a single word, the interpreter indicated the topic and established this as a starting point for the continuing story. Taking time to establish a common ground for the united focus (by establishing the topic “the altar’s painting”), the interpreter’s description prepared the recipient for the details to be introduced. The description was followed up by the deafblind person to use his own hands to make his own tactile impression by bodily interaction giving him the experience of being there at the site.

### Dialogical Approach

With Goffman’s terminology, the data reveal a dialogical approach in the ways the *frontstage* of the main conversation is made available (the group of deafblind guests meeting the guide and taking part in a guided tour) and in the ways important signals supporting the interaction and mediating process *backstage* (between the interpreter and the deafblind person) are performed. In the video of the guided tour, we searched for descriptions that provided information about other persons’ actions and the communicative situation, information that is crucial for dialog and interaction in the given context. Dialogical approaches are seen at both microlevel of adjustments in interaction and in a larger scale of dialogical involvement concerning the group.

**FIGURE 2:**
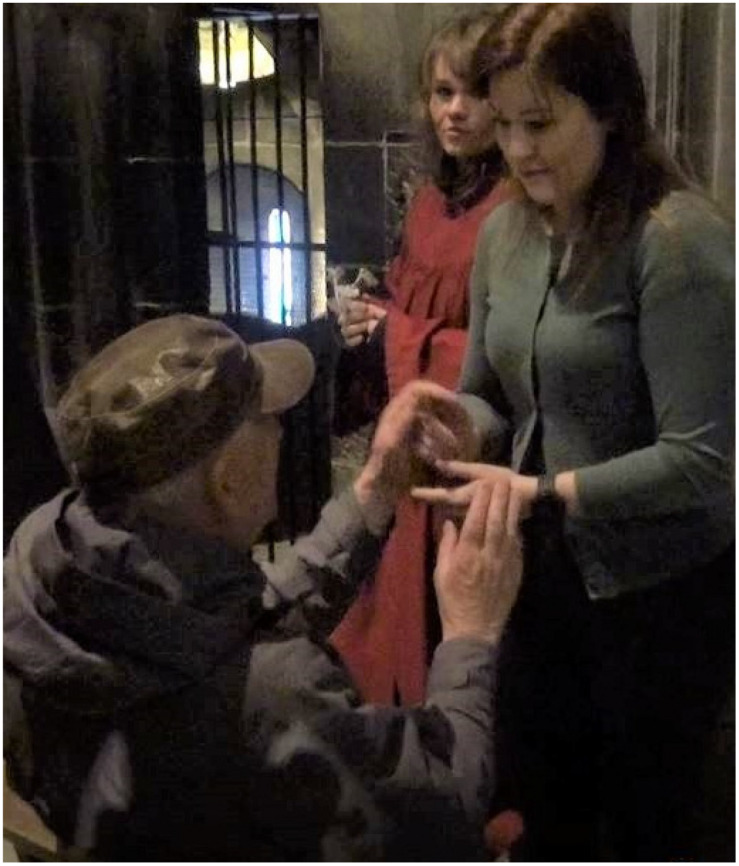
Tapping the interpreter’s hand as a response signal.

Figure 2 shows how communication is done in tactile modality which makes direct physical contact between the hands of the interlocutors. This hands-on contact opens for moment-by-moment dialogical interaction between the interlocutors. The deafblind person is sitting while the interpreter is standing, and during the hand-to-hand contact, response signals may be given from one participant simultaneously with the other’s utterances, opening for immediate dialogical adjustment and response. Here, the deafblind man is tapping on the interpreter’s hand with his right hand, as a signal of response. This interaction is a backstage activity relating to the process of understanding the interpreted utterance.

**FIGURE 3:**
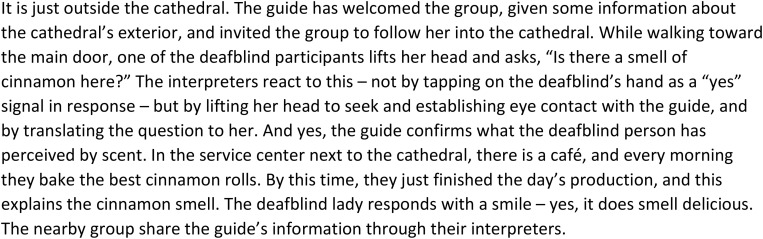
Building knowledge of the environment through one’s own inputs and questions.

Figure 3 provides an example of how one deafblind person’s utterances are established into the main conversation by the interpreter’s coordination of interaction between the deafblind person, the group, and the guide:

Being open to all kind of inputs that deafblind people receive in a situation from their own senses is here seen as a starting point for environmental description, linked to what the person him/herself has in focus. The way the interpreter coordinates and conveys the deafblind lady’s comment to the guide is an example of the dialogical approach, as is also seen in how the other interpreters coordinate the comments and questions to the other participants. It is essential to be a connecting link in communicative situations. It is not always possible for interpreters to find time to make all the necessary descriptions during an assignment. In Figure 3, the comment from the deafblind is translated directly by the interpreter, who makes an effort to establish contact with the guide and let the deafblind lady interact with her observation and comments. This leads to a communication sequence between the deafblind person and the guide. This comment becomes a part of the main conversation in the process where the deafblind person’s utterance is acknowledged by the interpreter, who establishes contact with the guide and addresses the question to her. The response from the guide becomes information to the rest of the group, and the input of environmental information becomes shared within the group. In the context of entering the cathedral, the information may seem to be less relevant. But this is not for the interpreter to “sort out” or quickly answer herself with a short response tapping at the deafblind’s arm. The question opens for several aspects of information and knowledge about the environment: Next to the cathedral, there is a service building, one that includes a café that in fact sells deliciously aromatic pastries, something that may come in handy after the tour. Thus, the interpreter’s open and dialogical awareness leads to additional information, involvement, and interaction. When the dialog also contains smiles and humor, this is also a useful additional reaction at the beginning of an activity where people are new to each other.

The guided tour includes several sections where the guide asks for comments from the group. The interpreters communicate questions, answers, comments, and reactions back and forth between all the participants, who are thus all free to participate in the situation.

**FIGURE 4:**
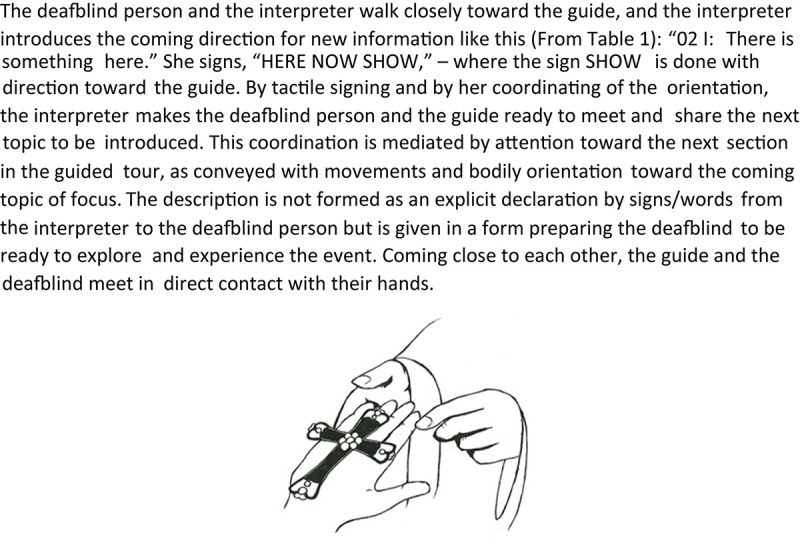
Information and focus between the guide and the deafblind participant.

**FIGURE 5:**
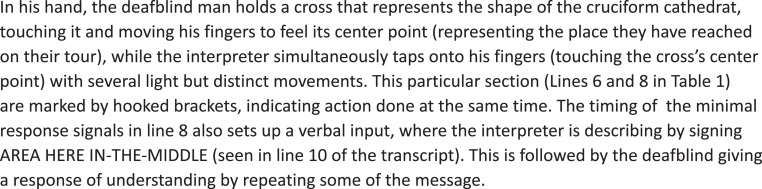
Description and backstage dialog.

The transcript in Figure 4 goes back to a part of the transcript in Table 1, where the interpreter indicates in what direction something will be in focus. This is an example of how interpreters work to provide direct information to the participants and let them experience things themselves, as seen in Figure 4:

Backstage activity continuously takes place in various forms, where short dialogs are handled without becoming part of the main conversation. In this study’s first transcript, in Table 1 (lines 04–11), we see examples of such a dialogical approach, as seen in Figure 5:

The dialogical form takes part in a sequence of involvement between the deafblind and the interpreter of the physical interaction, where the description process evolves between the deafblind and the interpreter and not being brought into the main conversation. The overlapping sections of tactile response in tactile communication are done similarly to the use of visual or auditive signals, such as nodding, smiling, or speaking, to respond and support a dialogical process in visual or auditive communicative modalities. Small signals, such as tapping lightly onto the interlocutor’s hands as a signal of confirming that a sign or a message is understood, were made throughout the event by the deafblind persons. The interpreters also frequently made such tapping response signals, when the deafblind repeated some of the interpreted message to ensure that it was perceived correctly. The example above is but one of many in our material of such tapping responses having the function of stating “yes,” “got it,” or “continue.” Those signals may be part of backstage or frontstage dialogs. Minimal response signals in the dialog process may also be a request for clarification. This may be done in a tactile way – holding the other’s hands lightly together to signal a wish to stop or to ask for a repetition of what was signed. Such approaches may also be seen on the microlevel of interaction, confirming the awareness and the speed of the communication process. Many of these signals support what may be seen as a backstage interaction that functions to confirm or adjust the interactive process between the pair of deafblind and interpreter – and are not brought into the main communication going on between the guide and group.

Moreover, at the microlevel, the coordination of the interaction between the deafblind person and the interpreter is dialogical.

**FIGURE 6:**
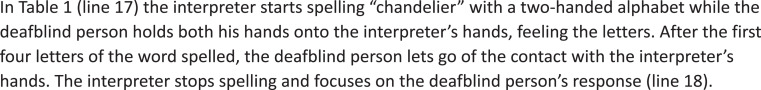
Backstage interaction between the interpreter and deafblind person.

When a deafblind person lets go of the interpreter’s hand as a word is being spelled, this is a signal, in this case, that the spelled fragment was enough for him to understand the meaning and that there was no need for further mediation of the word (Figure 6). This part of the interaction between their hands may be seen as a microbackstage interaction, where they negotiate to find an effective interaction between them. Having enough information to make meaning, this overlapping interaction makes the deafblind person ready to participate in the conversation. The comment from the deafblind (line 18) is handled as a contribution to the frontstage (the main dialog), and the interpreter brings the deafblind person’s response directly into the main conversation when she raises her voice and interprets his question to the guide (for her to answer). When this occurs, the other interpreters translate his comment into tactile sign language to be shared with the other members of the group as part of the shared frontstage focus. The shifts between backstage and frontstage activity run smoothly.

### Bodily Orientation

We will look more into examples of environmental description where the interpreter’s bodily orientation and moves add information about the given space and context. We have already discussed how such bodily orientation is part of the multimodal communicative tools closely linked to the context at hand.

**FIGURE 7:**
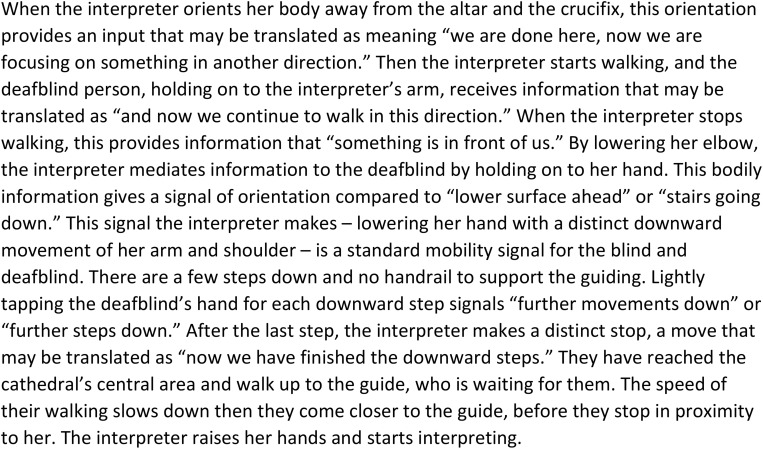
Bodily orientation and movements as a description.

**FIGURE 8:**
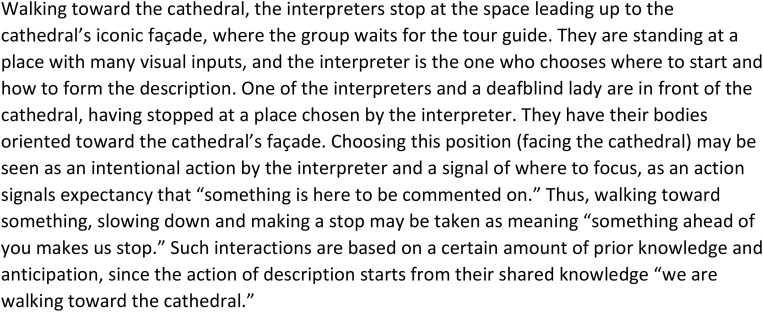
Bodily movements and positions approaching a site.

The next example relates to a situation before what happened in the transcript of Table 1. The cathedral’s guide has stopped by a huge silver crucifix standing on an altar close to the middle of the cathedral and presents some information. When the guide continues to the next stop on the tour, this means going to a new environment to learn even more. The interpreters primarily introduce this information through bodily movements and haptic signals in (Figure 7):

The annotated video data are transcribed in a narrative form where bodily movements present information about the current environment. The information is not expressed by words but by *bodily expression’s conveying a possible meaning potential* and as such translated into written text and formulated as above. A detailed analysis of the accessible parts of the interaction between the interpreter and the deafblind person reveals that these actions are part of the wide repertoire of multimodal communication tools in use, as in Figures 7 and 8.

Our data include this kind of environmental description where the interpreters make decisions on how to deliberately use their body and placement to provide access to the on-site interaction and environment. In the given context, the bodily movements provide multimodal information about the surroundings and add to the information provided by words or signs. The bodily actions the interpreter takes while walking are done in sensitive coordination with the guided deafblind person, inviting the other to follow his or her movements and adjusted if the deafblind seems insecure. The interpreter’s mode of description provides information and makes the deafblind’s orientation in a new environment safer.

Another example of bodily orientation as description comes from when the group is walking toward the cathedral and getting ready for the tour to start:

From the location in front of the cathedral, we will analyze the bodily orientation and shared knowledge that works together to form meaning. Stopping up enables both the deafblind person and the interpreter to start communicating.

**FIGURE 9:**
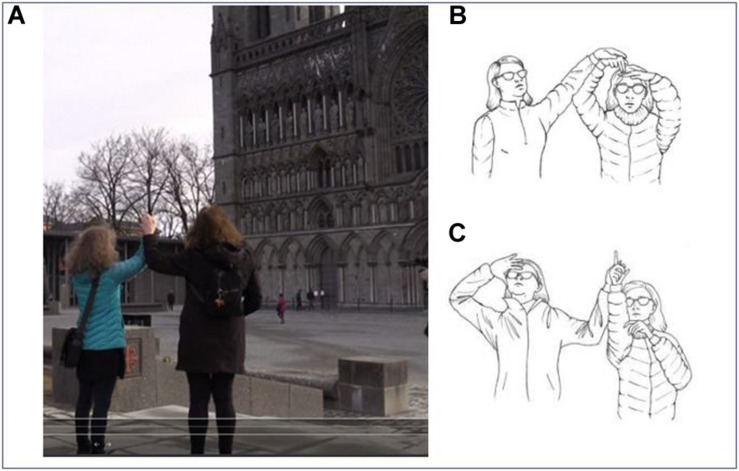
Description of the cathedral’s west side. **(A)** Overview **(B)** signing space **(C)** interaction in the description of the direction.

Stopping a slight distance away from the cathedral’s facade (as seen in Figure 9A), the interpreter gestures in the direction of the cathedral and then uses tactile signs to describe the high facade filled with sculptures. Having a distance to the cathedral makes it easier for the deafblind person (who has some limited vision) to get some impression of the building’s spectacular exterior described by the interpreter. For the interpreter, the placement is advantageous for taking in the cathedral and generating the description, with all the details present in front of her. Some part of the description is done in a dialogical way, through a combination of pointing movements and negotiations about where to look to find the spot to see. In Figure 9A, we see the interpreter and the deafblind person in the process of exploring some of the structures of the cathedral’s exterior, where the west facade is covered by rows of sculptures. Using signs high up in the signing space, articulated with the hands held high, the interpreter refers to the placement upon the facade. As we see in the pictures, the interpreter moves her hands in what is about the maximum possible height when she holds the other’s hand. Moving her hands to this extreme height, she presents the dimensions of the wall of sculptures (Figure 9B). With her eyes, the partially sighted deafblind lady (to the left in the drawings) seeks to take in some of this information. This is done in close interaction between the two of them, where the interpreter uses small hand movements to instruct the deafblind where to look. The deafblind’s head and eye movements are adjusted by the interpreter’s guiding and pointing. This joint process of steering and searching for possible visual information ends when the deafblind person gives a response. The dialogical adjustments between them are made through bodily movements and orientation (Figure 9C), and together they create a foundation for understanding the object in focus.

In this example, movement, orientation, holding hands, sign language, and pointing gestures are all communicative tools. The interpreter starts with a description of the dimensions of the cathedral, done by signing into an extended signing space, where the interpreter uses the maximum reach of her hands to express where the highest row of sculptures is located (Figure 9B). When the deafblind searches for where to look to see the start of the row, the interpreter points and uses her hands to steer the deafblind’s gaze (Figure 9C).

The sign language used (both outside the church and inside) contains conventional signs in tactile sign languages where sign language tools such as location, size, variation in movements, and intensity are seen during signing referring to activities or presenting artifacts at the site, as in Figure 9B, when the signing space is stretched up to refer to the highest row of sculptures of the wall.

## Discussion

### Dialogical Approach and Description

During the guided tour, we see that all the participants are active in the dialogical process. The guide’s contribution is clear: She invites the group to the site and leads the group through a program while she interacts with those involved and establishes the framework where the history will be told. Our data is from a tour dedicated to deafblind people to take part, and questions and comments from the participants are welcome. The guide interacts with the group and uses their feedback to provide more information to them (Mellemsether and Müller, 2016). The multimodal approach is seen in sequences of direct information given by touch, where the cathedral’s guide brings in models and artifacts to support the presentation of the cathedral’s form and special points of interest and sits with clear tactile landmarks to explore for the deafblind guest in the cathedral. The approach shows a multimodal understanding of interaction in the situation (Meisner et al., 2007; Mondada, 2016).

The interpreters are working in shifts to translate and provide an additional environmental description, adjusted to the context and the intention of those involved (Hjort, 2008; Raanes and Berge, 2011). The input to the descriptions is not a one-way activity from the sighted interpreters (and guide) to the deafblind group, as also initiatives from the deafblind participants may bring in new topics for further description and information. Each deafblind visitor and his or her interpreter shift their focus between the main frontstage conversations and their own backstage communication. There are simulations and actions of shifting focus toward the frontstage main conversations and backstage communication between the pair of deafblind and their interpreters (Goffman, 1971; Berge and Raanes, 2013). The dialogical approach supports the attention on to the main conversation and backstage.

Various kinds of techniques for environmental description are brought in by the interpreters, adjusted to the ongoing interaction and focus. The cathedral is a feature-rich environment with many detailed decorations and interiors. The interpreters combined a focus on this environment and a focus on the guide and the group’s actions and comments. In the large cathedral, there were other visitors present, and research assistants were on hand to film the interaction data from the deafblind. Information about this was given briefly at the beginning of the tour, but was later tuned down and not focused on by the interpreters or the group. This exemplifies how the interpreters actively select critical information based on a situated understanding of the event and how they focus on the main conversation (Metzger et al., 2004; Raanes, 2018). The information is focused on the cathedral, the guide, and the deafblind group, and the interpretation is done in a way that facilitates comments, questions, and a focus on the tour program. The dialogical frame is established based on a situated understanding of the situation and context (Wadensjö, 1995; Raanes, 2018). Simultaneously, the pairs of participants engage in many layers of brief, necessary clarification and cooperation at the backstage level (Goffman, 1981; Iwasaki et al., 2019).

### Multimodality in Interaction

The environmental description is provided with a wide repertoire of symbolic tools, including those studied in this article, namely bodily movement and orientation.

Tactile signing is an important communication method when interpreting to deafblind persons and may be adjusted to include descriptions in the way the signs are being performed (Holmström and Mesch, 2018). Tactile fingerspelling (and writing in the palm) is used when there are names, numbers, or words/signs to be presented. Haptic signals (that provide information on the deafblind person’s back or arms) and bodily signals, such as leading the deafblind person’s arms and sensitively exploring physical objects, are all methods in use to provide the environmental description. In our material, we also see that the deafblind persons are guided through the cathedral in a way that describes the various spaces, rooms, locations, and activities. This multimodal awareness is in line with other studies’ findings, such as Lahtinen et al. (2010), Edwards (2017), and Raanes (2020).

Our data also reveals the frequent use of tactile response signals as part of the communication. This is not a surprising finding. Several studies of deafblind conversations document the use of tactile response signals between the interlocutors (Willoughby et al., 2018, 2020). The tactile response signals are part of both the interpreter’s and the deafblind person’s contributions, whether supporting the main frontstage conversation or being a part of the backstage interaction between each deafblind person and their interpreter.

According to our observations, the interpreter’s attention toward body signals helps create awareness of when the other person seems ready for contact, ready to take in information, or ready to go on to new sections of information. This tactic seems incorporated in the interpreters studied here, as when an interpreter clarifies what the focus of attention should be in Table 1 when the interpreter walks toward the guide, who stands in the center point of the cathedral holding a model cross and looking at them. This may be understood as the interpreter taking the guide’s glancing at them as an invitation to come together, prompting the interpreter to lead the deafblind person over to the guide. The interpreter’s actions initiate and enable interaction between the guide and the deafblind person, as signaled through bodily movements and orientation. This awareness of a tactile orientation supports some of the findings in Edwards’ (2014) studies of the development of a pro-tactile movement in communities of American deafblind communities and in Gabarro-Lopez and Mesch’s (2020) study of interpreter-mediated action.

The use of space during tactile signing also supports the way that information is divided into sections, as when the interpreters in our data mark a previous topic by lowering their hands, which signals “this has ended” or “over to the next topic,” or when they transition to a new topic by slightly changing their bodily orientation before lifting their hands and introducing a keyword pointing to the coming topic. This kind of information by bodily movement and orientation was engaged by all participants. Initiatives to these actions function in a tactile way to signal the structures of the mediation process, analogous to the signals in interpreters’ work (The World Association of Sign Language Interpreters [WASLI], 2013; Napier et al., 2018).

The multimodal tools employed in descriptions are used with various techniques and focus on the intention and the critical information of the context. The multimodal information received by touch – such as focusing on the temperature differences in the different kinds of stone used near the main altar, or notions of variations of the floor done by one’s foot – are all part of the varied use of tactile and kinesthetic information. Sometimes, the guide or the interpreters make the deafblind group aware of such features, other times they are directly accessible by the deafblind person him- or herself. Information about the room and space may also be notified by the body, as when entering the cathedral’s tall and wide nave or coming into the narrow part at the inner altar. Some parts of Nidaros Cathedral include narrow arcades, where bodily information of air moving about when other visitors are passing may add to what the deafblind person notices. This supports a multimodal understanding of the interaction and demonstrates how multimodal resources, including language and bodily movements, add to the process of making meaning (Goodwin, 2007; Meisner et al., 2007; Mondada, 2016).

### Facilitating Environmental Description

As the analyzed data show us, tactile communication is a varied multimodal tool, and some forms of such communication require some extra time to be organized and performed clearly (Berge and Raanes, 2017).

In the analyzed example by the altar (Table 2), the interpreter uses fingerspelling and signs to introduce the concept “picture” for the altar’s copy of an old painting. Here, we see one possible reason for the repetition of signs and why the interpreter uses the combination of initial sign, fingerspelling, and sign seen in the transcript. The sign PICTURE has a form (the outline of a square) that may have more than one meaning that may be referring to more than one sign and concept. Since the deafblind person cannot see the mouthing of the word done by lip movements, the tactile signer is dependent on relaying just the manual part of the hands forming the sign. This may lead to a risk of misunderstanding. Since the sign PICTURE is part of the introduction of a new topic, the concept of what it refers to is not yet established. Presumably, signing PICTURE may not be precise enough to be understood. By offering additional fingerspelling of the word, the interpreter clarifies the topicalization in a varied, multimodal format. The interpreter takes the time to ensure the described message is received. Repetition and time are used to get the message across through various communication tools. All this is done in interaction with a clear focus on mutual awareness and coordination. A plan for organizing the information, where details are conveyed after an initial overview or a clarification of the given subject, is seen as a frequently used strategy. These are findings supported by data analyzed in the study by Raanes (2018) where topicalization is a way to mark what part a situated environmental description starts.

Being in the cathedral is to be at a pilgrimage site. Part of the interpreter’s (and the guide’s) multimodal awareness is to take time, when possible, to allow the deafblind guest to directly explore some of the features of this site. In such a strategy, the tour is to be explored and lived by the deafblind person as an active and primary participant in this activity, not a person whose role is to be passively informed. In practice, this means taking time to feel age-old marks on the cathedral’s wall made by stonemasons working on the cathedral hundreds of years ago, exploring an altar, feeling the form and temperature of different stones used in the structure, and so forth. The interpreters support the access to interaction within the group, as when the interpreters walk up to the guide and indicate that there is a feature of interest by signing “Here there is something” (Table 1, line 2); this is done by signing “HERE NOW SHOW,” where the sign SHOW is made in the direction of the guide. The guide is waiting and looking toward the deafblind person, and the interpreter understands this attention as an invitation to contact and walks close up to the guide. She adjusts her interpretation and description for a direct contact and involvement without being more proactive than necessary. Both the guide and the interpreters do so in the case described in Table 2; they keep their position and wait in order to allow such tactile activity by the deafblind visitor.

### An Extended Understanding of Communication Tools

This study provides new insights into the multimodal part of the interaction between the deafblind and their interpreters. The professional approaches from the field of interpreting for deafblind may be relevant and useful also in various personal and professional settings. The findings of multimodal communicative tools from this study may be relevant for extended target groups that work with and meet persons with blindness or dual sensory loss.

This study analyzes interpreters working on an environmental description for deafblind individuals. The situation does have some boundaries that may influence some of the choices the interpreters have to make. The way the situation is managed is affected by a program led by the guide of a tour of a cultural venue. Although this may predetermine many factors, we observed in our data that all the participants were in fact involved in the interaction. The guide does invite the visitors to explore the cathedral’s structure, pointing out touchable elements from various eras spanning the building’s almost thousand-year history, and to take part in discussions to find out more about historical and contemporary episodes from the cathedral.

The minimal dialogical clarifications found in the data are in many ways expected, as they are described in several studies of tactile signed conversation that these tactile signals are linked to the grip between hands during communication (Mesch, 2001; Raanes, 2006; Gabarro-Lopez and Mesch, 2020). What is new in this study is the finding that these signals play a role in opening for a dialogically influenced interaction during description. At every moment, the interpreters and the deafblind persons coordinate their interaction and show awareness of the other’s intention. It is important to be able to use interactional information as clues in a specific context and to build awareness *via* multimodal expressions and to recognize the expressions as meaningful in the interaction (Rommetveit, 1974; Trevarthen, 1998; Goodwin, 2011). This option is used both frontstage (concerning the main conversation) and backstage (supporting the cooperation between the interpreter and the deafblind person).

The ways that deafblind persons initiate turn taking by letting go of physical contact, thereby regulating the opportunity for tactile signing, are also a clear finding of how descriptions are negotiated. Such initiatives were seen during the interpreters’ signing or spelling of descriptions and serve to make the communication more effective – as when the meaning was understood and there was no need to continue the communicative input. Seeing these initiatives requires an in-depth analysis of all the variations of the communicative tools, as presented in Table 1. Variations in this regulation support the microlevels of communicative processes between the interlocutors. The awareness of these kinds of signals seems to be an important part of coordinating and interacting effectively in conversations in the tactile modality.

## Conclusion

This study is based on a small group of informants taking part in one specific communicative setting. From these limited data, the findings point toward how description may function as needed to empower the deafblind participants to make their own understanding of the context and the information they perceive. For interpreters and other professionals meeting persons with a dual sensory loss, it is important to have knowledge of the needs and techniques used to do environmental descriptions in a way that ensures critical information based on an understanding of the communicative setting and intention. The interpreter’s job description is often said to be neutral, establishing an invisible connection through non-selective mediation. Our study indicates that impartial description may be done in ways that is professional, ethically focused on the situated dynamic activity provided in a sequenced multimodal performance, and conducive to assisting the deafblind participate in meaningful interaction. Finds from this study point to the fact that providing description is deeply rooted in a situated understanding of context. Having time to focus on and build coherent contributions to both utterances and environmental information is of importance to participate and interact.

## Data Availability Statement

The raw data supporting the conclusions of this article will be made available by the authors, without undue reservation.

## Ethics Statement

The studies involving human participants were reviewed and approved by the Norwegian Centre for Research Data. The patients/participants provided their written informed consent to participate in this study – NSD 192998: Joint signing space in tactile sign language. The patients/participants provided their written informed consent to participate in this study. Written informed consent was obtained from the individual(s) for the publication of any potentially identifiable images or data included in this article.

## Author Contributions

The author confirms being the sole contributor of this work and has approved it for publication.

## Conflict of Interest

The author declares that the research was conducted in the absence of any commercial or financial relationships that could be construed as a potential conflict of interest.
